# Comparison of Transcriptional Response of C_3_ and C_4_ Plants to Drought Stress Using Meta-Analysis and Systems Biology Approach

**DOI:** 10.3389/fpls.2021.668736

**Published:** 2021-07-01

**Authors:** Ahmad Tahmasebi, Ali Niazi

**Affiliations:** Institute of Biotechnology, Shiraz University, Shiraz, Iran

**Keywords:** C_3_ and C_4_ plants, drought stress, meta-analysis, transcriptome, differentially co-expression

## Abstract

Drought stress affects a range of plant processes. It is still not well-known how C_3_ and C_4_ plants respond to drought. Here, we used a combination of meta-analysis and network analysis to compare the transcriptional responses of *Oryza sativa* (rice), a C_3_ plant, and *Zea mays* (maize), a C_4_ plant, to drought stress. The findings showed that drought stress changes the expression of genes and affects different mechanisms in the C_3_ and C_4_ plants. We identified several genes that were differentially expressed genes (DEGs) under stress conditions in both species, most of which are associated with photosynthesis, molecule metabolic process, and response to stress. Additionally, we observed that many DEGs physically located within the quantitative trait locus regions are associated with C isotope signature (d^13^C), photosynthetic gas exchange, and root characteristics traits. Through the gene co-expression and differential co-expression network methods, we identified sets of genes with similar and different behaviors among C_3_ and C_4_ plants during drought stress. This result indicates that mitogen-activated protein kinases (MAPK) signaling pathway plays an important part in the differences between the C_3_ and C_4_ species. The present study provides a better understanding of the mechanisms underlying the response of C_3_ and C_4_ plants to drought stress, which may useful for engineering drought tolerance in plants.

## Introduction

Climate change significantly limits the availability of water for plants and increases the intensity and frequency of drought periods (Parmesan and Hanley, 2015). As one of the major climate events, drought stress induces physiological and morphological changes, which can subsequently restrict the growth, yield, and quality of crops (Jump and Peñuelas, 2005). Under drought conditions, there are several adaptive mechanisms at molecular, cellular, and physiological levels in plants. Drought stress-induced responses lead to stress perception, signaling pathways, transcriptional alteration of genes, accumulation of osmotically active compounds and reactive oxygen species (ROS), extensive root system, and changes in the stomatal number, size, and architecture. In addition, water deficiency has an effect on the photosynthesis rate, carbon assimilation, stomatal conductance, and transpiration rate (Lawlor and Cornic, 2002; Harb et al., 2010; Lawas et al., 2018; Zhang et al., 2018).

The response of the plants to drought stress differs with the duration and intensity of stress, the species of plant, and their photosynthetic pathway. Despite the common reaction mechanism to water stress, significant differences in drought tolerance are observed among species. An important physiological factor influencing the response to drought is the difference between C_3_ and C_4_ photosynthesis (Hamim, 2005; Ghannoum, 2009; Taylor et al., 2014; Guidi et al., 2019). C_3_ and C_4_ plants with different modes of photosynthesis have evolved in different climates; therefore, they need different environmental conditions for optimal growth. Generally, C_4_ species are recognized as plants of warm and arid regions, while C_3_ species are of temperate origin. Accordingly, C_4_ species are expected to be better adapted to drought conditions than C_3_ species (Nayyar and Gupta, 2006).

C_4_ plants not only have a higher photosynthetic efficiency and CO_2_ fixation rates but also have a higher water use efficiency (WUE) and transpiration rate, which reflects their advantages compared to C_3_ plants. Photosynthetic activity in C_3_ and C_4_ species is significantly different under drought conditions. C_4_ species can effectively preserve high WUE under drought conditions, thus have a higher photosynthetic advantage than C_3_ plants (Taylor et al., 2011; Way et al., 2014; Hatfield and Dold, 2019).

In a comparative experiment, the drought limitation of photosynthesis between C_3_ and C_4_ grass species has been shown to be different. Recent studies have confirmed that gas exchange in the C_4_ plants was less affected by drought than that in the C_3_ plants (Yan et al., 2016). Some studies have reported that due to the photosynthetic advantages of C_4_ plants over C_3_ plants, in warmer and drier conditions, C_3_ plants can be replaced by C_4_ plants. However, it has been reported that C_4_ plants are more sensitive to soil water content than C_3_ plants with respect to their leaf carbon assimilation (Ripley et al., 2007; Wittmer et al., 2010; Labarrere et al., 2011; Luo et al., 2018; Zhong et al., 2019).

Advances in transcriptome sequencing have provided an opportunity to investigate simultaneous expression profiles of thousands of genes. A meta-analysis is an effective strategy to assess and combine different available transcriptome datasets. Importantly, a meta-analysis increases the statistical power, allowing the discovery of robust and reliable gene signatures. Integration of gene expression across species also is subject to determine conserved core gene sets and gene regulation evolution (Tseng et al., 2012; Shaar-Moshe et al., 2015; Tahmasebi et al., 2019).

Although meta-analysis has proven to be useful in discovering differentially expressed genes (DEGs), exploring relationships among genes is a critical step in predicting gene functions that can provide insight into biological processes. Gene co-expression network approaches use correlations between genes to cluster genes with similar expression profiles under multiple experimental conditions into co-expression modules. Gene co-expression modules reflect genes that contribute to the same biological pathways and processes. Such gene modules may be conserved across species and even across different kingdoms (Sibout et al., 2017). There are two strategies to compare co-expression networks in different species: First, identifying modules that are conserved across species with common gene orthologs, and second, identifying differentially co-expressed modules in which gene orthologs display different network structures between species.

The aim of this study is to compare the transcriptional response to drought stress from both C_3_ and C_4_ species to find important differences and similarities between them.

## Materials and Methods

### Data Collection, Preprocessing, and Meta-Analysis

Raw microarray expression data were retrieved from Gene Expression Omnibus^1^ and ArrayExpress.^2^ The species-specific array description (CDF) files and the corresponding probe annotations were downloaded from the Affymetrix site.^3^ The background correction and normalization of the expression data for each dataset were carried out using Robust Multichip Average (RMA) algorithm (Irizarry et al., 2003) within the Expression Console package. After preprocessing, to remove batch effects among different datasets, ComBat function in the SVA R package (Leek et al., 2012) was used based on an empirical Bayes method.

For each species, a meta-analysis was performed using the rank product statistics method to detect DEGs with RankProd package in R (Del Carratore et al., 2017). Genes with an FDR < 0.001 were considered as DEGs between the control and drought conditions.

To validate the results of meta-analysis, 10-fold cross-validation was used for expression values of DEGs in both species. In this validation approach, an initial dataset is split into a training set and a test set. One sample from the initial dataset is consecutively discarded for test and the others for training (Lorenzon et al., 2018; Tahmasebi et al., 2019).

To identify drought-response genes in other C_3_ and C_4_ species, two datasets (GSE48205 and GSE17669) that were composed of control and drought conditions were selected from the GEO database for sorghum (*Sorghum bicolor*) and barley (*Hordeum vulgare*). DEGs were identified using the GEO2R online analysis tool based on adjusted *p* < 0.05.

### Gene Ontology and Pathway Annotation of DEGs

Gene ontology (GO) of DEGs was implemented using the g: Profiler web tool (Reimand et al., 2016).^4^ The GO terms with adjusted *p* < 0.05 were considered to be significant terms. The important pathways were identified based on the Kyoto Encyclopedia of Genes and Genomes (KEGG) database.

### Determination of Orthologs

For species comparison, to distinguish predicted orthologs between *Oryza sativa* (rice) and *Zea mays* (maize), Model Genome Interrogator (MGI) tool in PLEXdb (Dash et al., 2012)^5^ and Ensembl (Hubbard et al., 2002) were used. The results from each method were combined into a nonredundant list of orthologous genes (Wang et al., 2014; Shaar-Moshe et al., 2015). Finally, all the identifiers were translated into rice locus ID.

### Consensus Network Analysis

To discover the common modules of C_3_ and C_4_, a weighted gene co-expression network analysis (WGCNA) consensus network was generated for the DEGs of the two species. Briefly, a similarity matrix [Sij = |0.5 + 0.5∗cor (xi, xj)|] was derived based on a Pearson correlation and transformed into an adjacency matrix [Aij = (|0.5 + 0.5∗cor (xi, xj)|)^β^] using a β of 12 as a soft-thresholding power. The adjacency matrix was converted into a topological overlap similarity measure (TOM), which was further used to obtain modules using the dynamic tree cut algorithm (Langfelder and Horvath, 2008) with a height of 0.25 and a deep split level of 2 and a minimum module size of 30. To determine the functions of modules, GO and KEGG enrichment analyses were performed for all the modules using g:Profiler web-based tool. Hub genes were determined based on high intramodular connectivity in the module.

### Differential Co-expression Analysis

DiffCoEx analysis method (Tesson et al., 2010) was used to identify differentially co-expressed modules between C_4_ and C_3_ based on WGCNA statistical framework, which finds gene sets with co-expression in one species but not in the other. In summary, an adjacency matrix within each species was built based on Pearson correlation for all pairs of genes and was used to compute the matrix of adjacency difference. A topological overlap matrix (TOM) was derived from the matrix of adjacency difference. Finally, differentially co-expression modules were detected by the “hybrid” method of dynamic tree cutting with a minimum module size of 30 genes. GO enrichment of modules was carried out with g:Profiler web-based tool.

### Co-localization Analysis of DEGs Against QTLs

To evaluate the co-localization of DEGs identified with reported quantitative trait loci (QTLs) for C isotope signature (d^13^C), photosynthetic gas exchange, drought tolerance, and root characteristics traits, we first obtained the genomic location of the QTLs based on the previous studies (Pelleschi et al., 2006; Takai et al., 2006; This et al., 2010; Gresset et al., 2014; Avramova et al., 2019) and Gramene database (Ni et al., 2009) and then compared the genomic coordinates of the DEGs with the QTLs. We retrieved the genomic sequences of the QTLs from the BioMart and aligned them with the sequences of the DEGs using Blastn (Woldesemayat et al., 2018) to identify the best blast hit with an E-values ≤ 1e-10 and identity >80%.

## Results

To determine which of the transcriptional responses were associated with drought stress, the meta-analysis was performed using the rank product approach for each species (Breitling et al., 2004). In total, 172 arrays corresponding to 11 drought stress studies, from two different plant species, were selected for the meta-analysis (Supplementary Table S1). Initially, we identified DEGs for each species separately. In total, the rice (as C_3_ plant) had 7,291 DEGs including 3,491 upregulated and 3,800 downregulated genes in drought compared to normal conditions (Supplementary Table S2). In the identified DEGs, probesets corresponding to *RAB16B* and *RAB21* genes were the most highly upregulated, while *PMEI-like* and *PEAMT2* genes were the most highly downregulated (Supplementary Table S2). Among the DEGs, some important genes such as *LEA*, *HSP70*, *WSI76*, and *DREB1C* were observed that play a role in stress tolerance. The maize (as C_4_ plant) had 4,915 DEGs with 2,532 upregulated and 2,383 downregulated genes in drought compared to normal conditions (Supplementary Table S2). In DEGs, probesets related to Cox family and fasciclin-like arabinogalactan proteins were the most highly upregulated and Histone H3-like proteins were the most highly downregulated. Among the DEGs, three genes encoding for drought-induced 19 (Di19) were upregulated under drought stress. Several genes encoding heat shock proteins were also detected among the DEGs. The 10-k fold cross was utilized to validate DEGs efficiency in distinguishing stress and control conditions. The result indicated that the control and stress samples were accurately classified, and the predictive accuracy for rice and maize was 98.72 and 97.22%, respectively.

**Figure 1:**
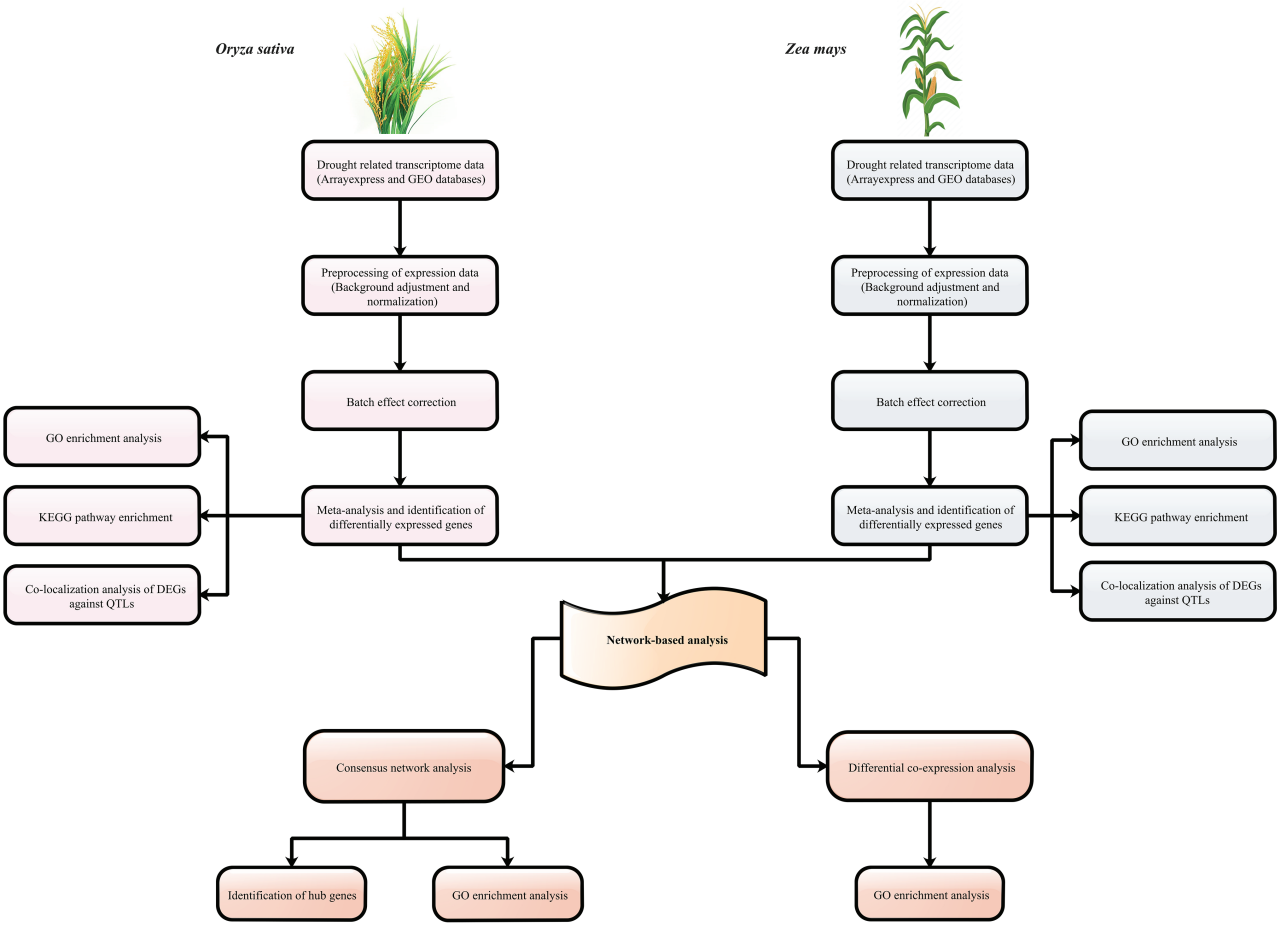
Schematic overview of the strategy for understanding the aspects of C_3_ and C_4_ plants to drought stress.

To further evaluate the results of meta-analysis, we used publicly available expression datasets for sorghum and barley. A total of 300 and 2,065 genes were found to be differentially expressed in sorghum and barley between the control and drought conditions, respectively (Supplementary Tables S3, S4). Additionally, the majority of DEGs were associated with alkaloid biosynthesis, plant hormone signal transduction, MAPK signaling pathway, response to abiotic stimulus, and carbon metabolism. Out of the DEGs detected in sorghum, a number of genes with transmembrane transporter activity were present, such as the SPX (Sb06g025950) and MS channel gene (Sb10g006710). In barley, we identified DEGs (Contig18416_at and Contig13030_s_at) that are mostly involved in the ABC transporter system. Among the total DEGs, 2 and 7.2% of sorghum and barely genes were orthologous with at least one of maize and rice DEGs, respectively. Among the DEGs shared between sorghum and maize, ASR protein was identified. ASR family may be expressed in under different conditions and shown to be involved in processes of plant development and in responses to abiotic stresses, such as water deficit, salt, and cold (Çakir et al., 2003). For the DEGs of barely that were orthologous with DEGs of rice, we found genes associated with the biosynthesis of secondary metabolites. A schematic workflow summarizing the major steps of this study is shown in Figure 1.

### Gene Ontology Enrichment Analysis in Each Species

**Figure 2:**
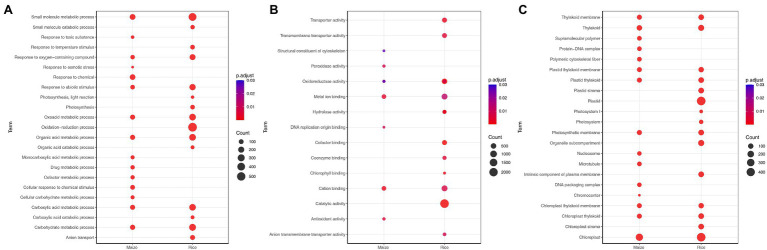
Gene ontology (GO) enrichment analysis of differentially expressed genes (DEGs) for maize (*Zea mays*) and rice (*Oryza sativa* L.), which shows the GO terms (adjusted *p* < 0.05) of biological processes **(A)**, molecular functions **(B)**, and cellular components **(C)** identified using g: Profiler. The size of the dot is based on the gene count enriched, and the color of the dot presents the terms enrichment significance.

To identify the functional characterization of significant DEGs in each species, GO analysis was conducted using g:Profiler tool. The top biological processes, which were significantly enriched in the rice, include photosynthesis, small molecule metabolic process, oxidation–reduction process, and response to abiotic stimulus (Figure 2A). Moreover, GO analysis highlighted the processes associated with plant hormone signal transduction (Supplementary Table S5). The most upregulated DEGs were enriched in response to temperature stimulus, response to salt stress, and response to osmotic stress, while the most downregulated DEGs were related to photosynthesis and light reaction (Figure 2B; Supplementary Table S5). In addition, GO term analysis based on molecular function was mainly associated with oxidoreductase activity and catalytic activity (Figure 2B). The most significant cellular component terms for DEGs were chloroplast and plastid (Figure 2C).

In maize, DEGs were significantly associated with small molecule metabolic process, response to chemical, carbohydrate metabolic process, and organic acid metabolic process (Figure 2A). For the upregulated DEGs, the most enriched GO term was response to osmotic stress, while the downregulated DEGs were enriched in photosynthesis and cofactor metabolic process. In the category of molecular function, cation binding, metal ion binding, and antioxidant activity were the top enriched GO terms among DEGs (Figure 2B). Meanwhile, the most significant cellular component terms for DEGs were DNA packaging complex, nucleosome, and thylakoid (Figure 2C). Notably, rice and maize had 34 and 25% of species-specific enriched biological processes, respectively. In addition, 41% of the terms were found to be common between the two plants (Supplementary Figure S1). The common biological processes were small molecule metabolic process and response to stress. Most of the genes associated with response to stress were also upregulated.

### Pathway Enrichment

**Figure 3:**
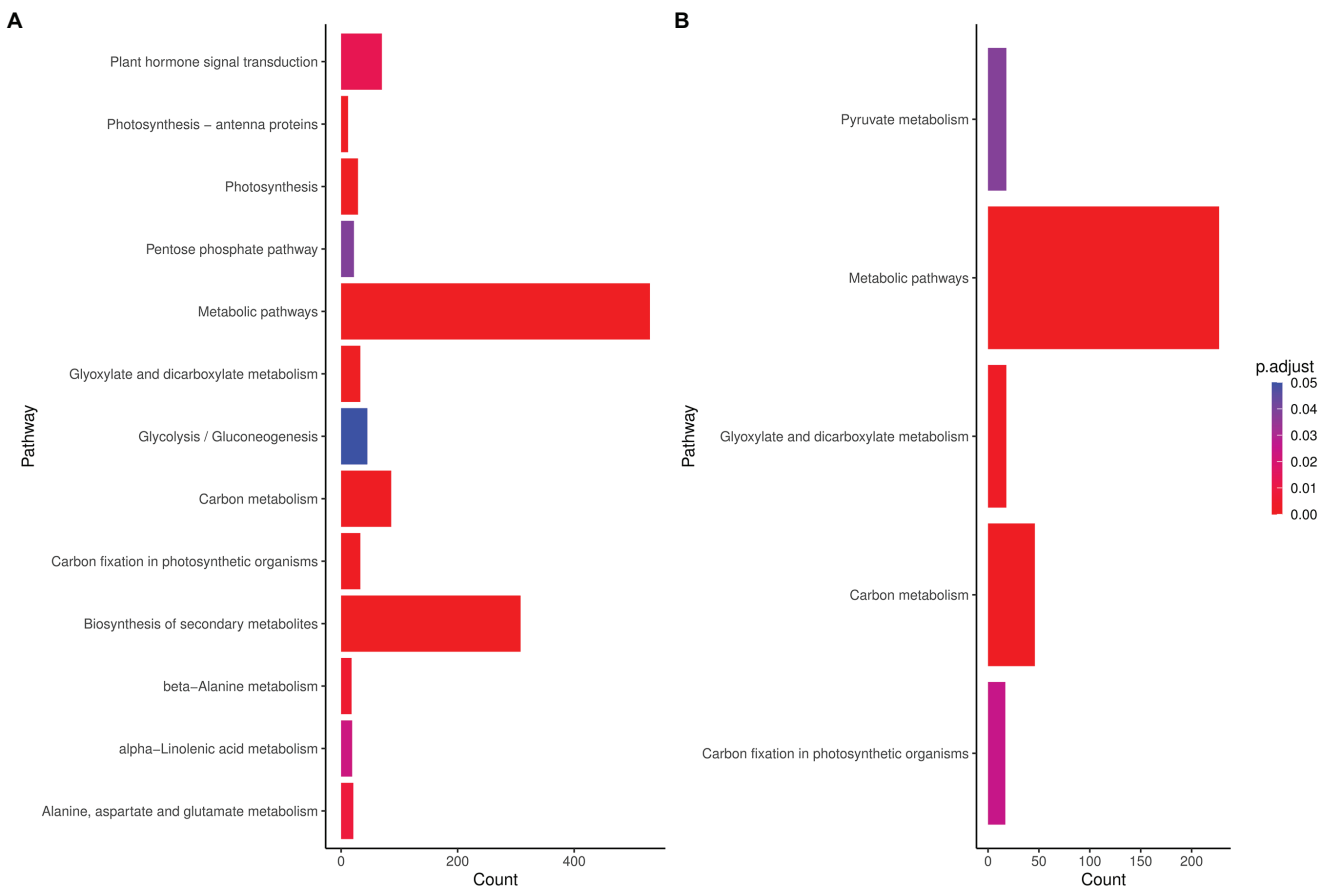
Kyoto Encyclopedia of Genes and Genomes (KEGG) pathways (FDR < 0.01) enriched for DEGs in **(A)** maize (*Z. mays*) and **(B)** rice (*O. sativa* L.). Count: Number of genes related to the enriched KEGG pathway. The color of the bar denotes adjusted *p*-value.

Through pathway analysis of DEGs obtained from the meta-analysis, we assessed and compared the pathways that might be associated with the response to drought stress in species. The results showed that metabolic pathways and carbon metabolism-related terms were enriched in maize, while among the 13 KEGG pathways identified in rice, metabolic pathways, photosynthesis, and biosynthesis of secondary metabolites were the most significant pathways (Figure 3). In rice, most of the genes related to the photosynthesis pathway and carbon fixation in photosynthesis were downregulated. The hormone signal transduction was also highly represented (Figure 3). The metabolic pathway was also significant in both plants and genes such as asparagine synthetase, acyl-CoA oxidase and peroxidases were up-regulated in this pathway. In addition, 1 and 9 pathways were unique in maize and rice, respectively (Figure 3).

### Identification of Consensus Modules

**Figure 4:**
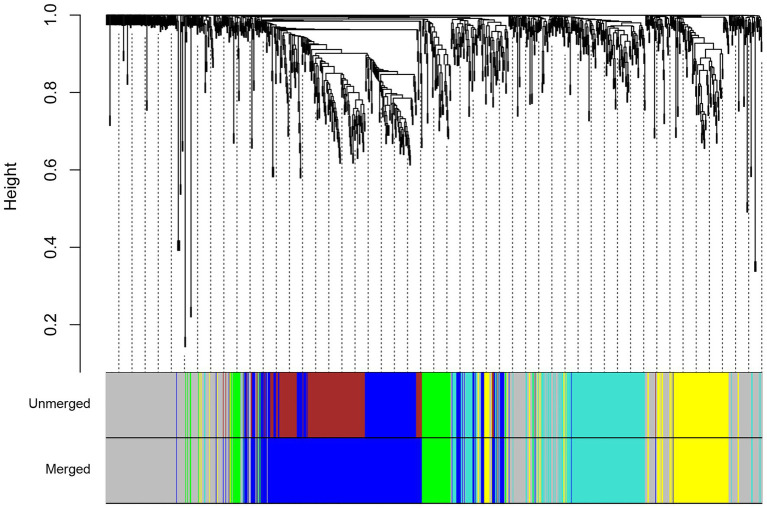
Hierarchical clustering tree for consensus modules identified by weighted gene co-expression network analysis (WGCNA). The color labels correspond to the different consensus modules identified between maize (*Z. mays*) and rice (*O. sativa* L.).

To generate the common networks and detect the conserved modules of genes with similar co-expression patterns in both species under drought stress, we performed a consensus network analysis. A total of four consensus modules were identified (Figure 4). Functional annotation showed that modules were associated with a wide range of functions (Supplementary Table S6). The turquoise module was enriched with genes related to response to water deprivation and small molecule metabolic process. The turquoise module had a number of bZIP and Myb transcription factor families, which suggests the significant role of this module in the regulation of drought stress responses in both species. The genes in the blue module were mainly enriched in cell wall organization and cell cycle, whereas the genes in the yellow module were mainly enriched in photosynthesis. In the green module, genes were significantly enriched in six GO biological process terms such as plant-type cell wall organization or biogenesis and reactive oxygen species metabolic process. The green module also contains genes involved in phenylpropanoid biosynthesis such as cinnamyl-alcohol dehydrogenase (CAD) and PRXs. Subsequently, the KEGG pathway analysis was performed to find pathway enrichment of genes in consensus modules. Among these modules, most of the genes were found to have been enriched in metabolic pathways; DNA replication; and valine, leucine, and isoleucine degradation (Supplementary Table S6).

#### Identification of Hub Genes

To discover the central and key genes related to the consensus modules, we determined genes with high connectivity within each module and selected as hub genes (Supplementary Table S7). The top two hub genes were *FOR1* and *PV72* for the blue module, *PDHE1-A* and *HyPRP18* for the green module, protein of unknown function *DUF676* and *PDX1* for the turquoise module, and ankyrin-like protein and *UBC37* for the yellow module eventually. The pathway analysis showed that the hub genes were mostly enriched in pyrimidine metabolism.

### Identification of Differential Co-expression Modules

**Figure 5:**
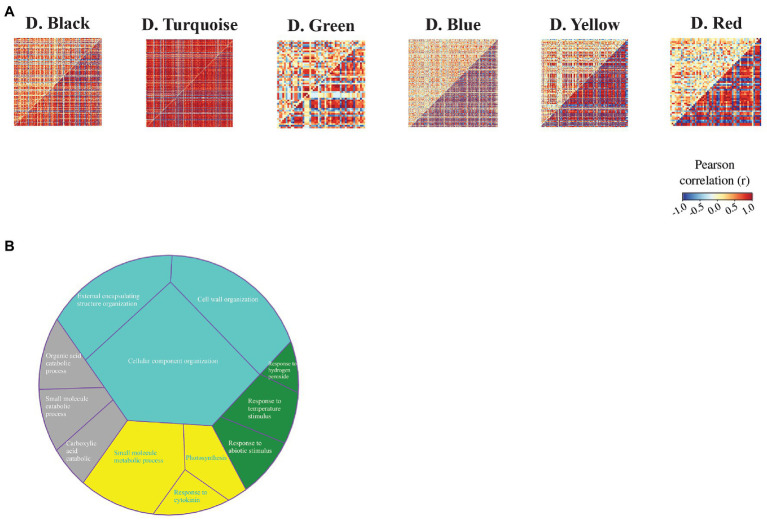
Differential co-expression modules identified between maize (*Z. mays*) and rice (*O. sativa* L.). **(A)** Heat maps display the correlation between all gene pairs contained within each module. The red and blue colors correspond to positive and negative correlations, respectively. **(B)** GO enrichment analysis of differential co-expression modules. The colors indicate differential co-expression modules.

To identify the sets of genes with differential co-expression patterns between the C_3_ and C_4_ species during drought stress, we used the DiffCoEx algorithm and constructed the differential co-expression network. In total, we obtained six modules of differential co-expression (Figure 5A). Functional enrichment results demonstrated that these modules were relevant to photosynthesis and response to cytokinin (D.yellow), organic acid catabolic process (D.black), response to stress (D.green), and cell wall organization (D.turquoise; Figure 5B). Additionally, the molecular function annotation indicated that the yellow module had a number of genes related to nitrate reductase (NADPH) activity. We also found that MAPK signaling pathway – plant (KEGG: 04016) and alanine, aspartate, and glutamate metabolism (KEGG: 00250) were enriched pathways among genes in red and blue modules, respectively (Supplementary Table S8).

### Co-localization of DEGs With QTL Intervals

Investigation of co-localization of DEGs with QTLs helps for determining the molecular genetic basis of important traits. In our study, several QTLs for drought tolerance, C isotope signature (d^13^C), photosynthetic gas exchange, and root characteristics traits were obtained from previous studies and Gramene database. We identified 1,724 and 801 DEGs for rice and maize, respectively, which were co-localized with QTLs (Supplementary Table S9). In rice, 122 (1.6%), 139 (1.9%), 105 (1.4%), and 1,358 (18.6%) DEGs localized within QTLs regions were associated with drought tolerance, photosynthetic gas exchange, d^13^C, and root characteristics traits, respectively. Moreover, in maize, 141 (2.8%), 444 (10.5%), 59 (1.2%), and 157 (3.19%) DEGs localized within QTLs regions were associated with drought tolerance, photosynthetic gas exchange, d^13^C, and root characteristics traits, respectively.

## Discussion

Drought stress responses significantly depend on the type of plant species. In C_3_ and C_4_ plants, stress results in several morphological, physiological, and molecular changes. It has been shown that the responses of C_3_ and C_4_ plants are distinct under drought conditions. Understanding the underlying mechanisms that generate differences is very important. In the present investigation, we have used meta-analysis and cross-species network analysis to identify the key genes and uncover similarities and differences in transcriptional response to drought stress between C_3_ and C_4_ plants.

In this study, by applying a rank product algorithm for meta-analysis, we were able to identify 4,915 and 7,291 DEGs under drought stress in maize and rice, respectively. Results of GO analysis revealed the functional categories of the DEGs in response to stimulus, metabolic pathways, and photosynthesis (Figure 2). In addition, it was observed that many DEGs that are associated with response to an abiotic stimulus such as heat shock protein, WRKY, histidine kinase, and alkaline alpha galactosidase 2 were upregulated, which might be correlated with water stress tolerance. Many genes involved in plant hormone signal transduction such as auxin-responsive protein, abscisic acid (ABA)-activated protein kinase, and jasmonate-induced resistance were also affected under stress conditions. The most highly upregulated gene in maize was *COX19-like* (Zm.5271.1.A1_at), a member of the ubiquitous COX (cytochrome c oxidase) protein family, which is involved in copper transfer in the intermembrane space of mitochondria for providing cellular energy (Bode et al., 2015; Radin et al., 2015). *RAB16B* (OS.51718.1.S1_AT), which belongs to the dehydrin protein family, was found as the most highly upregulated gene in rice. *RAB16B* plays an important role in drought tolerance, and its expression is regulated by ABA and osmotic stresses (Ono et al., 1996).

The classification of the shared orthologous DEGs into functional pathways suggests the involvement of these genes in the biosynthesis of secondary metabolites, glyoxylate and dicarboxylate metabolism, alanine metabolism, and carbon metabolism (Figure 3; Supplementary Table S2). However, the shared orthologous DEGs between rice and maize may indicate a different up or down direction. This suggests that these genes could represent the important aspect of the distinction between C_3_ and C_4_. *XTH17*, one of the DEGs with a different direction between the two plants, was downregulated in rice but upregulated in maize. XTH genes encode a class of enzymes that are associated with ethylene and regulate stress responses (Saab and Sachs, 1996; Song et al., 2018).

Based on the results, although the orthologous DEGs between the two plants were significantly overlapped, many of the DEGs were specifically expressed in maize and rice. For instance, *Pr1b* and *GST10* genes were differentially expressed only in maize. The *Pr1* gene is known as a pathogenesis-related protein and plays a key role in defense signaling pathways (Ali et al., 2018). In tomato, *Pr1* gene was upregulated in response to drought stress (Akbudak et al., 2020). GSTs are a group of cytoprotective enzymes participating in stress responses in plants (Kumar and Trivedi, 2018). Moreover, we investigated the co-localization of DEGs with known QTLs related to drought tolerance, d^13^C, photosynthetic gas exchange, and root characteristics traits in maize and rice and identified the DEGs that positioned under the QTLs. The results revealed that 23.6 and 16.2% of DEGs for maize and rice, respectively, were co-localized with these regions.

In maize, gibberellin receptor GID1L2 (ZM.8468.1.A1_AT) and ABA 8'-hydroxylases (ZM.9358.1.A1_AT) genes were located within the d^13^C QTL region. In addition, heat shock protein 90 (ZM.16505.1.A1_AT) and photosystem I reaction center subunit V (ZM.1085.2.A1_A_AT) were located between drought tolerance, photosynthetic gas exchange, and d^13^C QTL regions (Supplementary Table S9). These genes play key roles in the adaptive growth under stress conditions (Yang and Zeevaart, 2006; Xue et al., 2014; Yoshida et al., 2018) and will be considered as candidate genes associated with the QTLs of drought stress for future studies.

Although the meta-analysis focused on the individual genes, we employed consensus and differential co-expression analyses based on orthologous relationships to investigate the interactions among genes and discovered the conservation and differentiation of co-expression patterns in maize and rice under drought stress. By utilizing the consensus network analysis, we were able to identify conserved co-expression modules that could reveal common biological mechanisms in response to drought stress between two species. Four conserved modules were detected, including turquoise, blue, yellow, and green, that were highly involved in biological processes such as water deprivation, cell wall organization, and photosynthesis. In addition, the turquoise module was enriched for bZIP transcription factor genes. Previous reports suggested that the expression of Rubisco activase gene is regulated by bZIP transcription factors (Zhang et al., 2016). We also identified a number of transcription factors such as Myb, C_3_H, bHLH, MIKC, and YABBY that were conserved in both species. These transcription factors regulate genes involved in photosynthesis, the development of organs, and responses to environmental stimuli (Chang et al., 2012; Joshi et al., 2016). This observation suggests that a range of TF families participate in a regulatory network for drought response of the two photosynthetic types that are conserved. Two genes coding for plastid ribosomal proteins were observed in the yellow conserved module. These genes are associated with plastid translation, which is essential for cellular viability and plant development. It was confirmed that the chloroplast translation capacity is crucial to plant adaptation to stress, and its reduction has a direct effect on photosynthetic activity (Tiller and Bock, 2014; Pulido et al., 2018; Zoschke and Bock, 2018). This result indicates that plastid translation is a common mechanism under stress conditions for C_3_ and C_4_ plants.

The blue conserved module contains genes that have functions in DNA replication. Environmental stress leads to DNA damage in plants. The DNA repair process is a key mechanism for the maintenance of genome integrity. Previous reports implicated that the signaling mechanisms of the DNA damage response are strongly conserved in organisms (Yoshiyama et al., 2013; Nisa et al., 2019).

In addition, the green module included the PRX genes that have antioxidant activity and catalyze oxidoreduction between hydrogen peroxide and various reductants. It has been reported that PRX plays a critical role in multiple physiological processes by controlling hormonal metabolism and antioxidant defense (Hiraga et al., 2001; Jouili et al., 2011). Moreover, we screened out the hub gene, PDHE1-A, from the green module. PDHE1 plays an important role in the auxin conjugate sensitivity and auxin transport (Thelen et al., 1999).

According to the pathway analysis on the genes within modules (Figure 5), we found that pathways were significantly enriched in the suberine and wax biosynthesis, valine, leucine and isoleucine degradation, photosynthesis pathway, and phenylpropanoid biosynthesis. The phenylpropanoid biosynthetic pathway is one of the major secondary metabolite routes involved in the biosynthesis of plant phenolics. Phenolic accumulation is a defensive mechanism for multiple environmental stresses (Thelen et al., 1999). Additionally, several genes with unannotated genes are co-expressed within turquoise and green modules, which suggests the genes may be associated with stress adaptation.

We also performed a differential co-expression analysis to investigate alterations in the co-expression patterns of DEGs between C_3_ and C_4_ species, which provides information about pairs of DEGs connected in C_3_ but not in C_4_. We used the DiffCoEx algorithm and compared the expression patterns of orthologous DEGs in rice and maize. We identified five modules (including yellow, black, green, turquoise, red, and blue), which contained genes with differential co-expression between species under drought conditions. These genes are mainly associated with processes of response to stress, metabolic pathways, and photosynthesis. We found genes related to the hormone cytokinin in the yellow module. This module contains known genes of plant hormone signal transduction, such as *RR2*, *RR4*, *RR9*, and *RR10*. These genes have a central role in cytokinin-mediated functions, affecting processes such as growth, development, and response to various abiotic stress. Studies in rice (*O. sativa*) have also shown that different RR genes impress photosynthesis genes (Wang et al., 2019). These results indicated that RR genes could be key genes for the different responses between C_3_ and C_4_ plants to drought stress. The D.yellow module was enriched for the key carbon fixation genes (*PEPC2*, *GADPH*, *PRK*, *FBA*, and *SBP*) that play fundamental roles in photosynthesis genes that have different response patterns between C_3_ and C_4_ plants under drought stress. In addition, reticulon-like protein was observed in the yellow module. Plant reticulons are considered to be essential in endoplasmic reticulum and contribute to trafficking pathways (Lee et al., 2011; Kriechbaumer et al., 2015).

The results of enrichment analysis showed that DEGs in the green module were mainly associated with response to stress. Interestingly, a gene encoding transcription factor ethylene insensitive 3 (EIN3), which is associated with MAPK signaling pathway was present in the red module. This gene participates in the signal transduction network and plant immunity (Chen et al., 2009).

In addition, heat shock protein-encoding genes were in the green module, indicating that the response of heat shock proteins to drought stress is one of the major differences between C_3_ and C_4_ species. The blue red module also contains HMGR gene, which regulates the synthesis of terpenoids. Expression changes of HMGR correlate with adaptation to demanding environmental conditions (Zhang et al., 2020). This suggests that this gene can be important for adaptive capacity to stress in plants.

## Conclusion

The photosynthetic characteristics are an important aspect in response to stress. We used meta-analysis and co-expression network analysis to compare the response of C_3_ and C_4_ plants against drought stress. The meta-analysis identified the key genes associated with response to drought for C_4_ and C_3_ plants. The results indicated that drought influences a wide range of biological processes in both plants. Here, we demonstrate that many of the DEGs co-localize with the previously identified drought-QTLs. The findings highlight several differences and similarities that exist between the two types of plants, such as the small molecule metabolic process, photosynthesis, response to cytokinin, and response to stress. Moreover, the results strengthen the association between MAPK signaling pathway and differences between the C_3_ and C_4_ species in response to drought stress. We also identified RR and EIN3 genes as putative genetics targets for engineering drought tolerance between C_4_ and C_3_ plants.

## Data Availability Statement

The datasets presented in this study can be found in online repositories. The names of the repository/repositories and accession number(s) can be found in the article/Supplementary Material.

## Author Contributions

All authors contributed equally to this work and approved it for publication.

### Conflict of Interest

The authors declare that the research was conducted in the absence of any commercial or financial relationships that could be construed as a potential conflict of interest.

## Footnotes

^1^http://www.ncbi.nlm.nih.gov/geo

^2^http://www.ebi.ac.uk/arrayexpress

^3^http://www.affymetrix.com

^4^https://biit.cs.ut.ee/gprofiler/

^5^http://www.plexdb.org

## Supplementary Material

Supplementary Figure S1 Venn diagram of specific and common significantly enriched biological processes terms in maize (*Zea mays*) and rice (*Oryza sativa* L.).
