# Supplementary figures and images for: Comparison of Transcriptional Response of C3 and C4 Plants to Drought Stress Using Meta-Analysis and Systems Biology Approach
Source: Front Plant Sci. 2021 Jul 1;12:668736. doi: 10.3389/fpls.2021.668736 (PMC8280774; doi:10.3389/fpls.2021.668736)

**Oryza sativa**

**Zea mays**

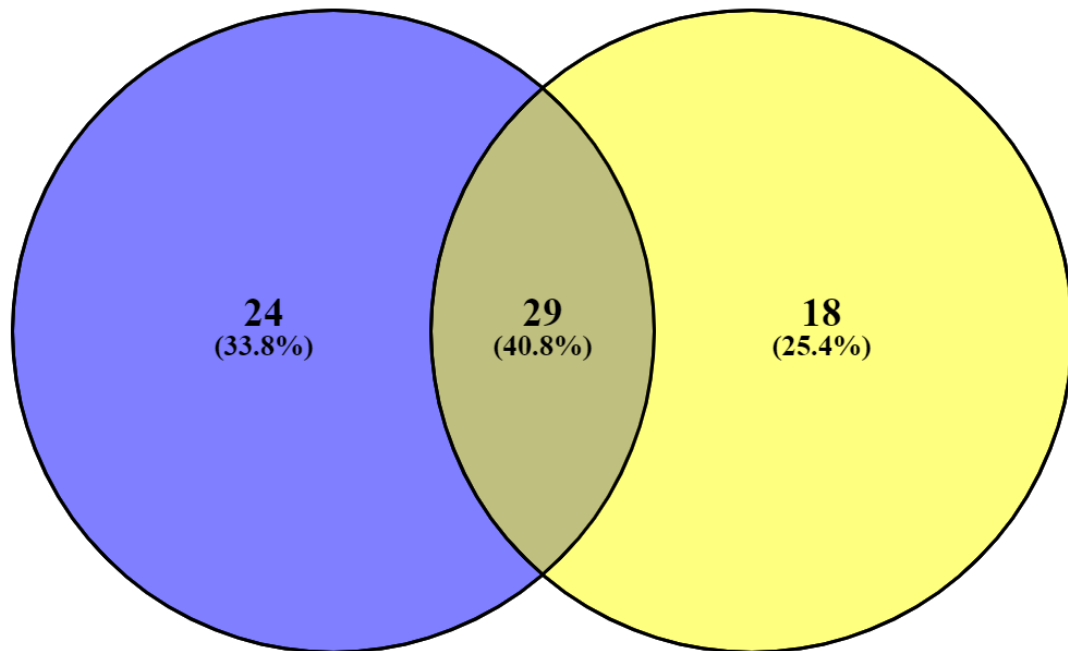

Supplement: Supplementary Figure S1 — Venn diagram of specific and common significantly enriched biological processes terms in maize (Zea mays) and rice (Oryza sativa L.). [file Data_Sheet_1.PDF]
